# Rapid Diagnostic Tests for the Detection of the Four Dengue Virus Serotypes in Clinically Relevant Matrices

**DOI:** 10.1128/spectrum.02796-22

**Published:** 2023-01-23

**Authors:** Nina M. Pollak, Malin Olsson, Madeeha Ahmed, Javier Tan, George Lim, Yin Xiang Setoh, Judith Chui Ching Wong, Yee Ling Lai, Jody Hobson-Peters, Joanne Macdonald, David McMillan

**Affiliations:** a Centre for Bioinnovation, University of the Sunshine Coast, Sippy Downs, Queensland, Australia; b DMTC Ltd., Kew, Victoria, Australia; c School of Science, Technology and Engineering, University of the Sunshine Coast, Sippy Downs, Queensland, Australia; d Environmental Health Institute, National Environment Agency, Singapore, Singapore; e Yong Loo Lin School of Medicine, National University of Singapore, Singapore, Singapore; f School of Chemistry and Molecular Biosciences, The University of Queensland, St. Lucia, Queensland, Australia; g BioCifer Pty. Ltd., Brisbane, Queensland, Australia; Emory University School of Medicine

**Keywords:** dengue virus, NS5 gene, rapid sample preparation, recombinase polymerase amplification, isothermal amplification, lateral flow detection, rapid molecular assays

## Abstract

The efficient and accurate diagnosis of dengue, a major mosquito-borne disease, is of primary importance for clinical care, surveillance, and outbreak control. The identification of specific dengue virus serotype 1 (DENV-1) to DENV-4 can help in understanding the transmission dynamics and spread of dengue disease. The four rapid low-resource serotype-specific dengue tests use a simple sample preparation reagent followed by reverse transcription-isothermal recombinase polymerase amplification (RT-RPA) combined with lateral flow detection (LFD) technology. Results are obtained directly from clinical sample matrices in 35 min, requiring only a heating block and pipettes for liquid handling. In addition, we demonstrate that the rapid sample preparation step inactivates DENV, improving laboratory safety. Human plasma and serum were spiked with DENV, and DENV was detected with analytical sensitivities of 333 to 22,500 median tissue culture infectious doses (TCID_50_)/mL. The analytical sensitivities in blood were 94,000 to 333,000 TCID_50_/mL. Analytical specificity testing confirmed that each test could detect multiple serotype-specific strains but did not respond to strains of other serotypes, closely related flaviviruses, or chikungunya virus. Clinical testing on 80 human serum samples demonstrated test specificities of between 94 and 100%, with a DENV-2 test sensitivity of 100%, detecting down to 0.004 PFU/μL, similar to the sensitivity of the PCR test; the other DENV tests detected down to 0.03 to 10.9 PFU/μL. Collectively, our data suggest that some of our rapid dengue serotyping tests provide a potential alternative to conventional labor-intensive RT-quantitative PCR (RT-qPCR) detection, which requires expensive thermal cycling instrumentation, technical expertise, and prolonged testing times. Our tests provide performance and speed without compromising specificity in human plasma and serum and could become promising tools for the detection of high DENV loads in resource-limited settings.

**IMPORTANCE** The efficient and accurate diagnosis of dengue, a major mosquito-borne disease, is of primary importance for clinical care, surveillance, and outbreak control. This study describes the evaluation of four rapid low-resource serotype-specific dengue tests for the detection of specific DENV serotypes in clinical sample matrices. The tests use a simple sample preparation reagent followed by reverse transcription-isothermal recombinase polymerase amplification (RT-RPA) combined with lateral flow detection (LFD) technology. These tests have several advantages compared to RT-qPCR detection, such as a simple workflow, rapid sample processing and turnaround times (35 min from sample preparation to detection), minimal equipment needs, and improved laboratory safety through the inactivation of the virus during the sample preparation step. The low-resource formats of these rapid dengue serotyping tests have the potential to support effective dengue disease surveillance and enhance the diagnostic testing capacity in resource-limited countries with both endemic dengue and intense coronavirus disease 2019 (COVID-19) transmission.

## INTRODUCTION

Globally, the incidence of dengue has increased 30-fold over the past 50 years (1), with more than half of the world’s population now at risk of DENV infection (2). The disease is caused by infection with one of four serotypes of dengue virus (DENV). The immune responses developed against one DENV serotype fail to provide long-lasting immunity against the remaining three serotypes. Secondary infection with a different DENV serotype is also associated with more severe disease, a phenomenon known as the antibody-dependent enhancement phenomenon (3, 4), which is attributed to the cocirculation of different serotypes in the same geographical region (5). The lack of access to quality diagnostics remains a major contributor to the dengue health burden in resource-limited settings. Rapid and accurate diagnostics for the detection and differentiation of DENV serotypes are urgently needed. Of special interest are DENV point-of-care diagnostics for clinical care, surveillance, and outbreak control. An ideal DENV point-of-care test is of low cost and easy to use while being well suited for widespread screening according to the ASSURED (affordable, sensitive, specific, user-friendly, rapid, robust, equipment-free, and deliverable to end users) criteria for diagnostic testing (6, 7).

The early and accurate serotyping of DENV is made challenging by several factors, including a limited time window for detection, varying low viral loads, and insufficient laboratory resources in low-income countries with limited health care resources (8, 9). While nucleic acid-based assays can detect viruses earlier in the infection cycle than serology-based assays, the current gold standard, reverse transcription-quantitative PCR (RT-qPCR), requires laboratories due to the difficulties in preparing samples and the time-consuming nature of testing. Rapid antigen tests such as the NS1 rapid diagnostic (RD) test can have reasonable sensitivity (71 to 80%) and good specificity for the detection of dengue infection in general (100%) but do not discriminate serotypes (10). A novel anti-NS2BNS3pro antibody-based indirect enzyme-linked immunosorbent assay (ELISA) showed good sensitivity (87%) and reasonable specificity (76%) (11). Diagnostics based on the CRISPR/Cas13 system allow the fast, highly sensitive, and specific detection of DENV (12–15). Among new technologies for clinical diagnostics, reverse transcription-recombinase polymerase amplification (RT-RPA) does not require thermal cycling instrumentation for nucleic acid amplification, showing the potential to provide rapid, low-resource detection that can be adapted for serotype-specific detection (16–19). RPA can be combined with lateral flow detection (LFD) technology to require minimal equipment suitable for resource-limited settings (20–23). However, both RT-qPCR and RT-RPA require purified RNA, which must be obtained using specialized equipment and trained personnel. The current global coronavirus disease 2019 (COVID-19) pandemic has illustrated how standard RNA extraction kits can become a bottleneck for testing, not only due to its time-consuming and labor-intensive nature but also because of unprecedented supply issues. Clinical diagnosis of suspected dengue cases could also greatly benefit from the elimination of RNA extraction kits, particularly where a lack of infrastructure exists, and excludes the use of costly automated robotics systems.

We previously developed four rapid serotype-specific DENV tests that employed RT-RPA-LFD assays that discriminate among the four DENV serotypes using a simple low-resource format that requires only a heating block and pipettes for liquid handling. One of the key novelties of these tests was the use of a one-step sample processing step for RNA preparation that is suitable for use in low-resource settings (24). In the current study, we evaluated the ability of our tests, including one-step sample processing, to detect dengue virus RNA from clinically relevant sample matrices and clinical samples. Our results show that the one-step sample preparation method inactivated DENV present in these matrices and enabled detection at clinically relevant sensitivities. These results enabled the development of a protocol to apply the four rapid tests for the detection of DENV in blood, plasma, and serum samples.

## RESULTS

### Analytical sensitivities and specificities of rapid serotype-specific DENV RT-RPA-LFD assays using purified RNA.

**FIG 1:**
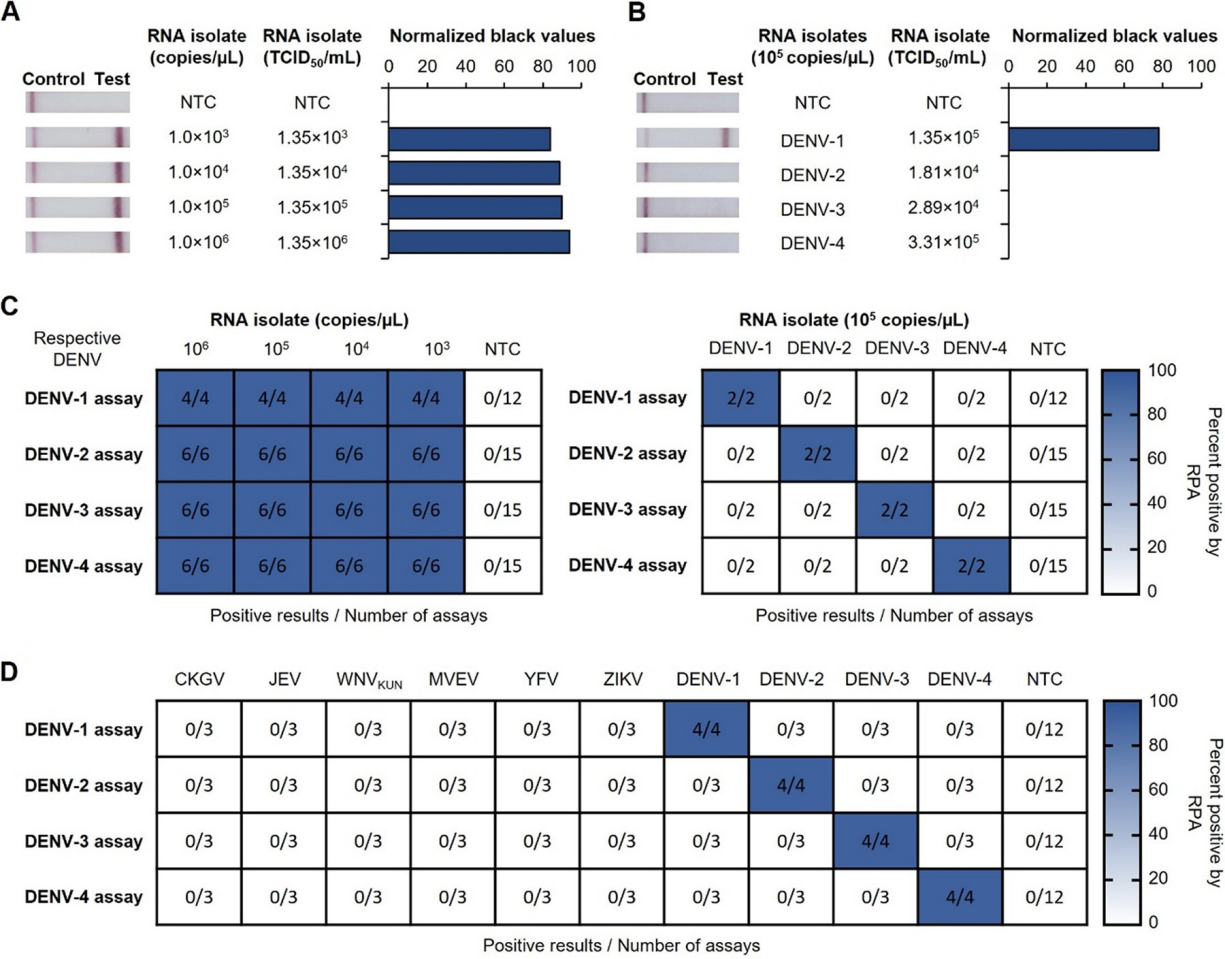
Analytical sensitivities and serotype specificities of the four serotype-specific DENV RT-RPA-LFD assays using kit-purified DENV isolate RNA. (A) Sensitivity testing used purified RNA from a DENV-1 isolate (ET00.243). (B) Serotype specificity was trialed with purified RNA from DENV-1 (ET00.243), DENV-2 (New Guinea C), DENV-3 (ET00.209), and DENV-4 (ET00.288) isolates at 10^5^ copies/μL as determined by universal dengue RT-qPCR. (A and B, left) Photographs of lateral flow strips with control bands (all samples) and test bands (positive samples). Nuclease-free water was used as a no-template control (NTC). (A and B, right) Normalized pixel densities (black values) from the test displayed. (C) Heat maps summarizing serotype-specific DENV RT-RPA-LFD assay results for the detection of purified DENV-1, -2, -3, and -4 RNAs, with the corresponding isolates displaying sensitivity (left) and serotype specificity (right) data. (D) Heat map summarizing RT-RPA-LFD test results for the detection of DENV-1, -2, -3, and -4, testing RNA isolated from chikungunya virus (CKGV), Japanese encephalitis virus (JEV), West Nile virus subtype Kunjin (WNV_KUN_), Murray Valley encephalitis virus (MVEV), yellow fever virus (YFV), and Zika virus (ZIKV), with DENV-1, -2, -3, and -4 RNA transcripts as the positive controls at 10^3^ copies/μL in the corresponding assays. Nuclease-free water was used as NTC for all testing.

We first confirmed the analytical sensitivity of our four serotype-specific DENV RT-RPA-LFD assays using kit-purified viral RNA from DENV isolates produced in the Aedes albopictus mosquito cell line C6/36. Positive results were observed in a range from 1 × 10^6^ to 1,000 copies/μL for all four dengue assays (Fig. 1A and C; see also Fig. S1A, C, and E in the supplemental material). Serotype-specific analytical specificities were subsequently confirmed by conducting all four assays using kit-purified DENV-1 to -4 RNAs; each assay showed a positive result only for the serotype that it was designed to detect (Fig. 1B and C). To assess strain specificity, serotype-specific DENV RT-RPA-LFD assays were tested against multiple strains of the same serotype. These DENV-2 (ET300, PR159, and Timor 2004) and DENV-3 (Cairns 2008) strains were successfully detected by their respective assays, with detection limits ranging from 10 to 1,000 copies/μL of TRIzol-purified RNA (Fig. S2). Analytical specificities were also confirmed by trialing each assay for the detection of TRIzol-purified RNA from other mosquito-borne flaviviruses as well as the alphavirus chikungunya virus (CHIKV), with each assay detecting only viral RNA of its own serotype and none of the other viral RNAs trialed (Fig. 1D; Fig. S3).

### Detection and inactivation of cultured DENV-1 to -4 using TNA-Cifer reagent E.

**FIG 2:**
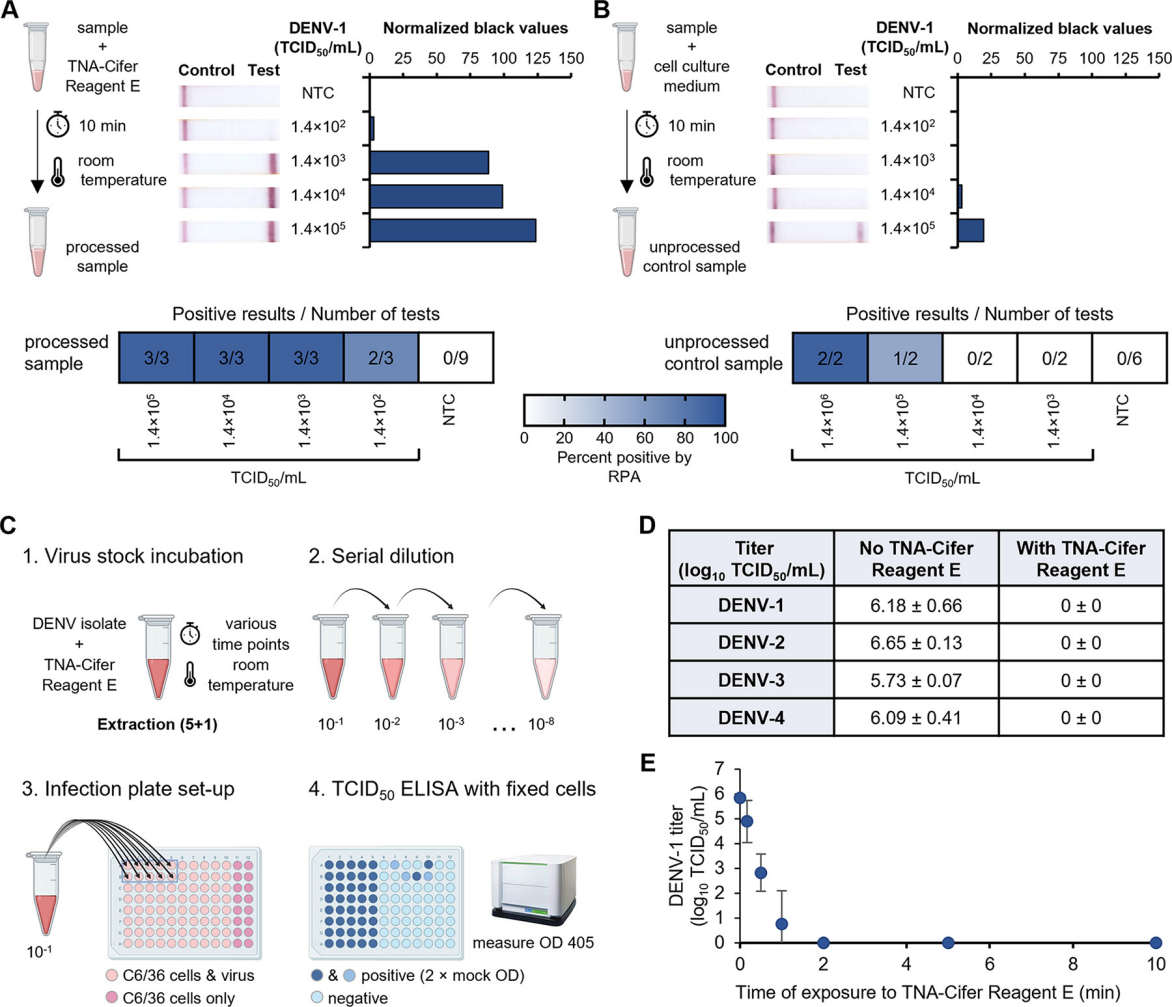
Detection and inactivation of cultured DENV-1 to -4 using TNA-Cifer reagent E. (A and B) TNA-Cifer reagent E (A) or cell culture medium (B) was added to dilutions of a DENV-1 isolate (ET00.243) in a 5:1 ratio (sample to TNA-Cifer reagent E or cell culture medium), incubated for 10 min at room temperature, and tested for detection with the rapid DENV-1 serotyping test. (A and B, left) Sample preparation diagrams; (middle) photographs of lateral flow strips with control bands (all samples) and test bands (positive samples). (A and B, right) Normalized pixel densities (black values) from the test displayed; (bottom) heat map summarizing RT-RPA-LFD test results. Cell culture medium was used as the no-template control (NTC). (C) Workflow of inactivation testing performed with TNA-Cifer reagent E and DENV-1, including virus stock incubation (1), serial dilution (2), infection plate setup (3), and a TCID_50_ ELISA with fixed cells (4). OD, optical density. (D) TNA-Cifer reagent E inactivation of DENV-1 (ET00.243), DENV-2 (New Guinea C), DENV-3 (ET00.209), and DENV-4 (ET00.288) after a 10-min incubation at room temperature at a 5:1 ratio. Averages ± standard deviations are shown (*n* = 3). (E) Virus titer after DENV-1 (ET00.243) was incubated with TNA-Cifer reagent E at a 5:1 ratio (i.e., 100 μL of virus in cell culture medium added to 20 μL of TNA-Cifer reagent E) for 0, 0.167, 0.5, 1, 2, 5, and 10 min. This experiment was performed independently three times, with one sample taken per time point; the average results ± standard deviations are displayed.

Our rapid dengue diagnostic tests combine a rapid sample preparation step utilizing TNA-Cifer reagent E (BioCifer Pty. Ltd.) with serotype-specific DENV RT-RPA-LFD assays. To determine if our rapid preparation step was viable, the full diagnostic test was evaluated using cultured DENV. Virus was detected in the range of 1,400 to 14,000 50% tissue culture infectious doses (TCID_50_)/mL within 35 min (Fig. 2A; Fig. S4) for all four serotypes. In the absence of TNA-Cifer reagent E, the test band intensities were greatly diminished (Fig. 2B).

Interestingly, we observed that TNA-Cifer reagent E inactivated DENV after a 10-min incubation at room temperature (RT) (Fig. 2C and D). Further investigation revealed that complete DENV-1 inactivation occurred in as early as 2 min if 5 parts sample was mixed with 1 part TNA-Cifer reagent E (Fig. 2E). DENV-1 was also inactivated if 1 or 2 parts sample was mixed with 1 part TNA-Cifer reagent E (Table S1). Together, these results show that TNA-Cifer reagent E was capable of inactivating DENV across multiple concentrations of TNA-Cifer reagent E.

### Optimization of rapid dengue serotyping tests for clinically relevant mock samples.

**FIG 3:**
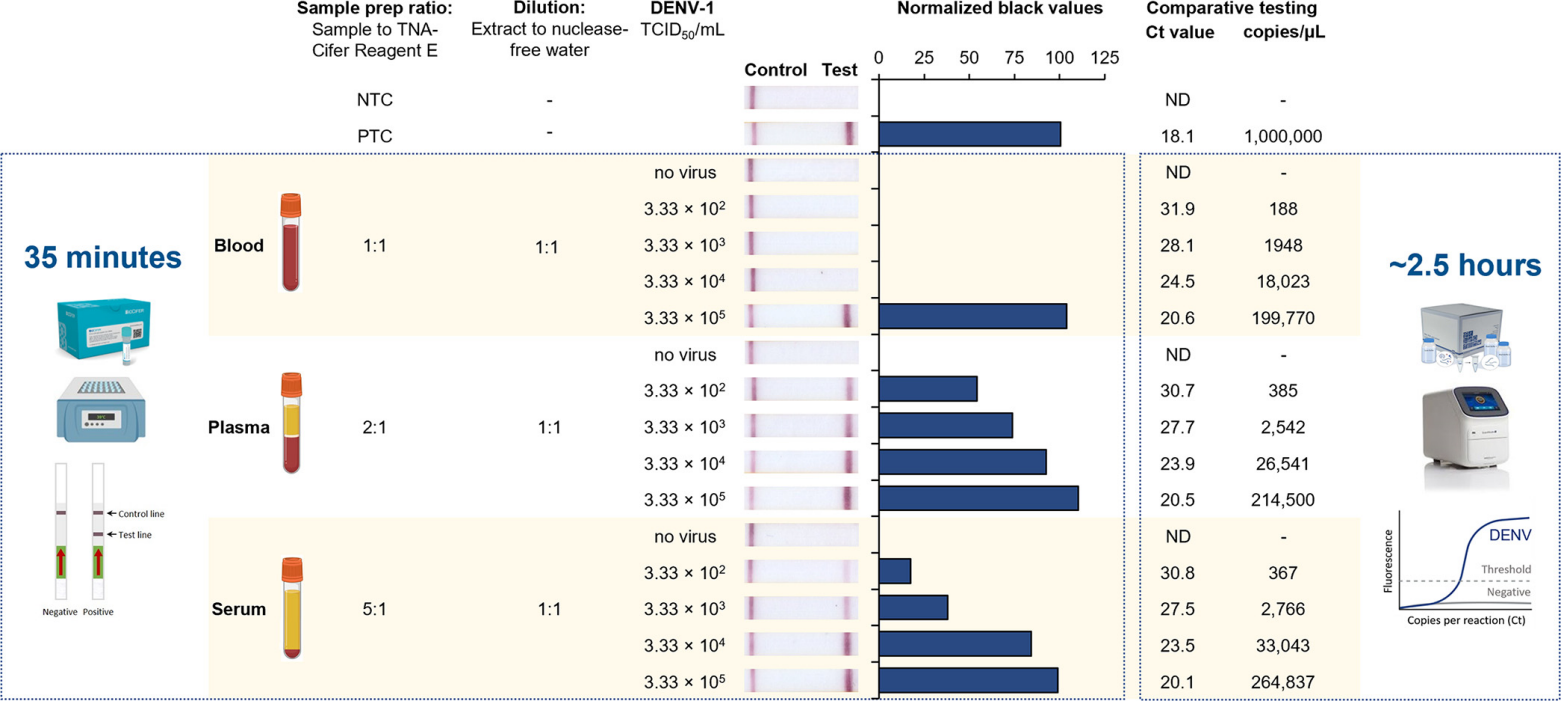
Optimized rapid DENV-1 serotyping test detection of DENV-1 in human blood, plasma, and serum spiked with a DENV-1 isolate. (Left box) Cartoon displaying the simplified procedure, with the time consumed (left); sensitivity testing using human K_3_ EDTA blood, plasma, or serum containing dilutions of cultured DENV-1 (ET00.243) (second from the left); sample preparation (sample prep) and dilution ratios (middle); a photograph of lateral flow strips with control bands (all samples) and test bands (positive samples) (second from the right); and normalized pixel densities (black values) from the test displayed (right). (Right box) Comparative RT-qPCR cycle threshold (*C_T_*) values and copy numbers (copies per microliter) (left) and cartoon illustrating the simplified methodology with the time consumed (right). (Top) Nuclease-free water was used as the no-template control (NTC), and transcribed RNA of DENV-1 was used as the positive control (PTC) at 10^6^ copies/μL. Blood, plasma, and serum without the spiked DENV-1 isolate were used as negative controls (no virus). ND, not detected.

We next assessed if TNA-Cifer reagent E was compatible when viral RNA samples were derived from clinically relevant matrices. This was achieved by spiking high-titer DENV into serum, plasma, and blood. For each of the samples, the ratio of the sample to TNA-Cifer reagent E and the subsequent dilution step were first optimized, resulting in matrix-specific protocols for serum, plasma, and blood (Table S2 and Fig. S5). To assess the limit of detection using these optimized protocols, first dilutions of DENV-1 (ET00.243) were either (i) prepared in blood, plasma, and serum and trialed using our rapid DENV-1 serotyping test protocol or (ii) purified for detection using universal dengue RT-qPCR. Our rapid DENV-1 diagnostic test detected a minimum of 333 TCID_50_/mL in mock plasma and serum samples, which was calculated by RT-qPCR to contain between 367 and 385 RNA copies/μL (Fig. 3). Blood did interfere with rapid sample processing and testing, however, with the lowest DENV-1 titer detected being 333,000 TCID_50_/mL (199,700 RNA copies/μL). We subsequently evaluated additional mock samples spiked with other DENV strains and serotypes and confirmed the successful detection of DENV in blood to 94,000 to 330,000 TCID_50_/mL (Fig. S6A), in plasma to 940 to 22,500 TCID_50_/mL (Fig. S6B), and in serum to 3,330 to 22,500 TCID_50_/mL (Fig. S6C).

### Clinical sensitivity and specificity of the rapid serotype-specific tests using clinical serum samples.

**FIG 4:**
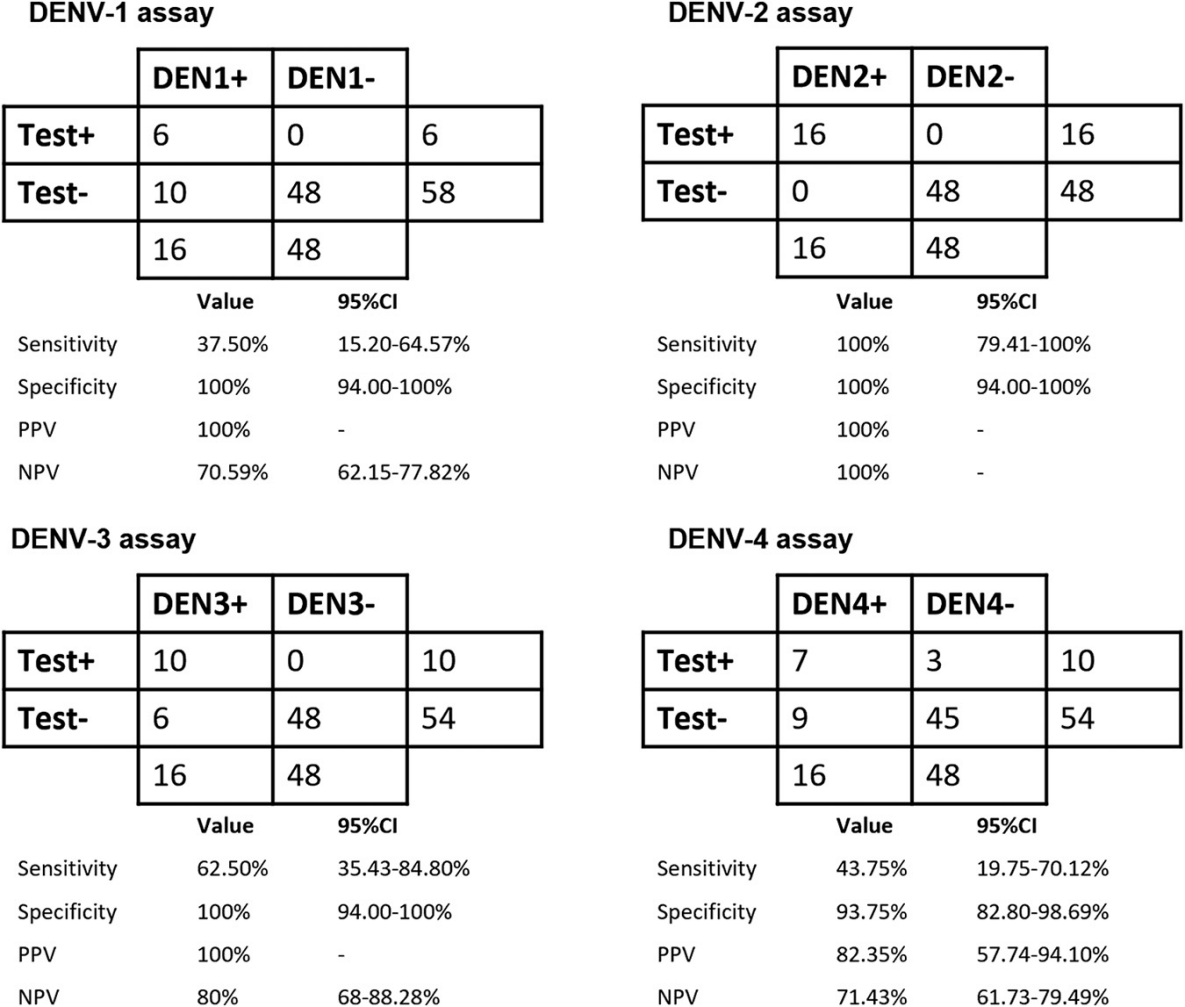
DENV serotype test performance characteristics in relation to DENV serotyping RT-qPCR as a reference. Test performances for each serotype are presented as 2-by-2 tables. As this was a stratified sample set, positive predictive values (PPV) and negative predictive values (NPV) were calculated based on an assumed dengue prevalence of 40%. (Raw data are available in Table S3 in the supplemental material.)

We then trialed the rapid serotype-specific tests using 80 archived patient serum samples (DENV-1-positive sera, *n* = 16; DENV-2-positive sera, *n* = 16; DENV-3-positive sera, *n* = 16; DENV-4-positive sera, *n* = 16; DENV-negative sera, *n* = 16). The respective DENV serotype-specific assays were able to successfully detect serotype-positive sera with test specificities of between 94% (confidence interval [CI], 82 to 94% [61/64 serum samples]) and 100% (CI, 94 to 100% [64/64 serum samples]). Among the assays, the DENV-2 serotyping test yielded the highest test sensitivity of 100% (CI, 80 to 100% [16/16 serum samples]), while the DENV-1, DENV-3, and DENV-4 assays yielded test sensitivities of 38% (CI, 15 to 65% [6/16 serum samples]), 63% (CI, 35 to 85% [10/16 serum samples]), and 44% (CI, 20 to 70% [7/16 serum samples]), respectively (Fig. 4; Table S3).

## DISCUSSION

Across tropical and subtropical regions of Asia, dengue fever accounts for approximately 70% of the global disease burden (25). Diagnostics discriminating among DENV serotypes are particularly important due to antibody-dependent enhancement of viral infection (3), a phenomenon in which virus-specific antibodies can promote rather than inhibit disease. The four dengue virus serotypes (DENV-1, -2, -3, and -4) pose substantial challenges to health care systems, particularly in resource-limited countries with both endemic dengue and intense COVID-19 transmission (26). In the present study, we demonstrated that four rapid serotype-specific DENV RT-RPA-LFD diagnostic assays developed for the detection of infection in mosquitoes (24) could be adapted for the detection of DENV in mock human blood, plasma, and serum samples using a similar one-step sample preparation method. Our rapid dengue serotyping tests are therefore suitable for application in low-resource clinical settings to detect dengue viral RNA in the acute-illness phase, requiring only a heating block and pipettes for liquid handling. We demonstrated that each serotype-specific DENV RT-RPA-LFD assay had excellent specificity, detecting only the serotype-specific target, and did not detect any other DENV serotypes or other common arboviruses. The four assays were shown to detect as few as 10 to 1,000 copies/μL purified RNA, and the rapid test format directly detected cultured DENV within 35 min, without the requirement for the separate purification of RNA. Analytical sensitivity testing demonstrated the detection of as few as 1,400 to 14,000 TCID_50_/mL, equivalent to approximately 1 to 14 infectious particles/μL.

Most RPA-based DENV diagnostic studies have used real-time RT-RPA in conjunction with an Exo fluorescence probe rather than an Nfo probe, which is more commonly used for LFD (16–19). Two real-time RT-RPA Exo assays targeting the 3′ untranslated region (UTR) have previously been described for the independent detection of DENV-1 to -3 and DENV-4. These assays had analytical sensitivities similar to those of our tests (100 and 10 RNA copies/μL, respectively). When trialed clinically, these assays also demonstrated 98% sensitivity (*n* = 31) and 100% specificity (*n* = 23) for the detection of DENV in plasma. A field trial in Bangkok, Thailand, showed that the clinical sensitivity and specificity were 72% (*n* = 90) and 100% (*n* = 41), respectively (17). Another study reported a universal dengue RT-RPA Exo assay similarly targeting the 3′ UTR with a detection limit of 10 RNA copies/μL and clinical sensitivity and specificity of 77.0% and 97.9%, respectively, in serum samples (*n* = 203) (16). The kit was then converted for LFD by exchanging the Exo probe with an Nfo probe, with clinical testing (*n* = 120) demonstrating 100% concordance with RT-qPCR results (21). More recently, another recombinase-based isothermal amplification technology has emerged, known as recombinase-aided amplification (RAA). A universal dengue RT-RAA-LFD assay targeting the 3′ UTR was demonstrated to have a detection limit of 10 copies/μL and clinical detection in serum samples (*n* = 247) with sensitivity and specificity of 98.92% and 100%, respectively, compared to RT-qPCR (27). All of these studies, however, required the purification of RNA prior to RT-RPA and did not enable strain serotyping. Our four rapid dengue serotyping tests (with Nfo probes for LFD) showed analytical sensitivities similar to the ones reported in those previous studies, without any requirement for RNA purification, detecting DENV-1 to -4 in both plasma and serum to as few as 333 to 22,500 TCID_50_/mL. For blood, our DENV-1 to -4 tests were not as sensitive. The isolation and detection of viral RNA in blood are challenging due to the unstable nature of viral RNA and the presence of immunoglobulin G and enzyme-inhibiting compounds such as anticoagulants, heme, and RNase, with the latter degrading RNA. In the present study, we demonstrated that viral RNA can be detected reliably and reproducibly with our fast sample-processing protocol using serum and plasma. All four assays successfully detected serotype-positive clinical samples with test specificities of between 94 and 100%. While the DENV-2 assay yielded a test sensitivity of 100% and could detect DENV-2 up to 0.004 PFU/μL (equivalent to the sensitivity of PCR), the other DENV assays had limited test sensitivities of 38 to 63%, with the lowest detected values being between 0.03 and 10.9 PFU/μL. Hence, further improvements are necessary to enhance the test sensitivities of the DENV-1, DENV-3, and DENV-4 assays. One possible consideration could be to align the nucleotide binding regions of the primer/probe sequences with those of circulating DENV strains.

The requirement for sample preparation has been a challenge for the development of nucleic acid amplification tests (NAATs) at the point-of-care. Current assays and platforms often require lengthy procedures and multiple instruments to remove contaminants and inhibitors from clinically relevant, complex samples (28). Our study is the first to describe a simple one-step sample preparation method that inactivates DENV from blood, plasma, and serum. Prepared samples can be directly used for isothermal amplification after a dilution step. Importantly, our data indicate that the virus was inactivated in the first step of the procedure, which is critical for the safety of operators in pathology laboratories and for reducing the requirements for biosafety level 2 (BSL-II) biosafety cabinets. TNA-Cifer reagent E could thus provide a pathway for safer point-of-care testing or safer testing in low-resource environments that do not have full biohazard facilities. Furthermore, the simple workflow for the rapid dengue serotyping tests could eliminate the need for the transportation of samples from primary health and district centers to well-equipped reference centers/laboratories for RNA isolation (29). Further studies testing TNA-Cifer reagent E inactivation of known blood contaminants, such as human immunodeficiency virus (HIV) and hepatitis B virus, could help ensure the full safety of operators at the point of care.

LFD-based point-of-care devices are rapidly emerging strategies for qualitative and quantitative analysis allowing accurate readout by eye by minimally trained personnel (8). One current limitation of RPA-LFD for point-of-care use is its restriction to singleplex detection. We envision that our four serotype-specific DENV RT-RPA assays could be combined in a single-tube reaction coupled with multiplexed LFD through the use of additional 5′ molecular labels with suitable detection antibodies to allow discrimination between targets (30–32).

In conclusion, our rapid dengue serotyping tests were able to detect as few as 1,400 to 14,000 TCID_50_/mL in rapidly processed samples, omitting the need for traditional RNA purification. Our tests have several advantages compared to RT-qPCR, such as a simple workflow, rapid sample processing and turnaround times (35 min from sample preparation to detection), minimal equipment needs, and improved laboratory safety through the inactivation of the virus during the sample preparation step. The low-resource format of our rapid dengue serotyping tests could help ensure effective dengue disease surveillance and enhance the diagnostic testing capacity in low-resource settings.

## MATERIALS AND METHODS

### Study design.

**FIG 5:**
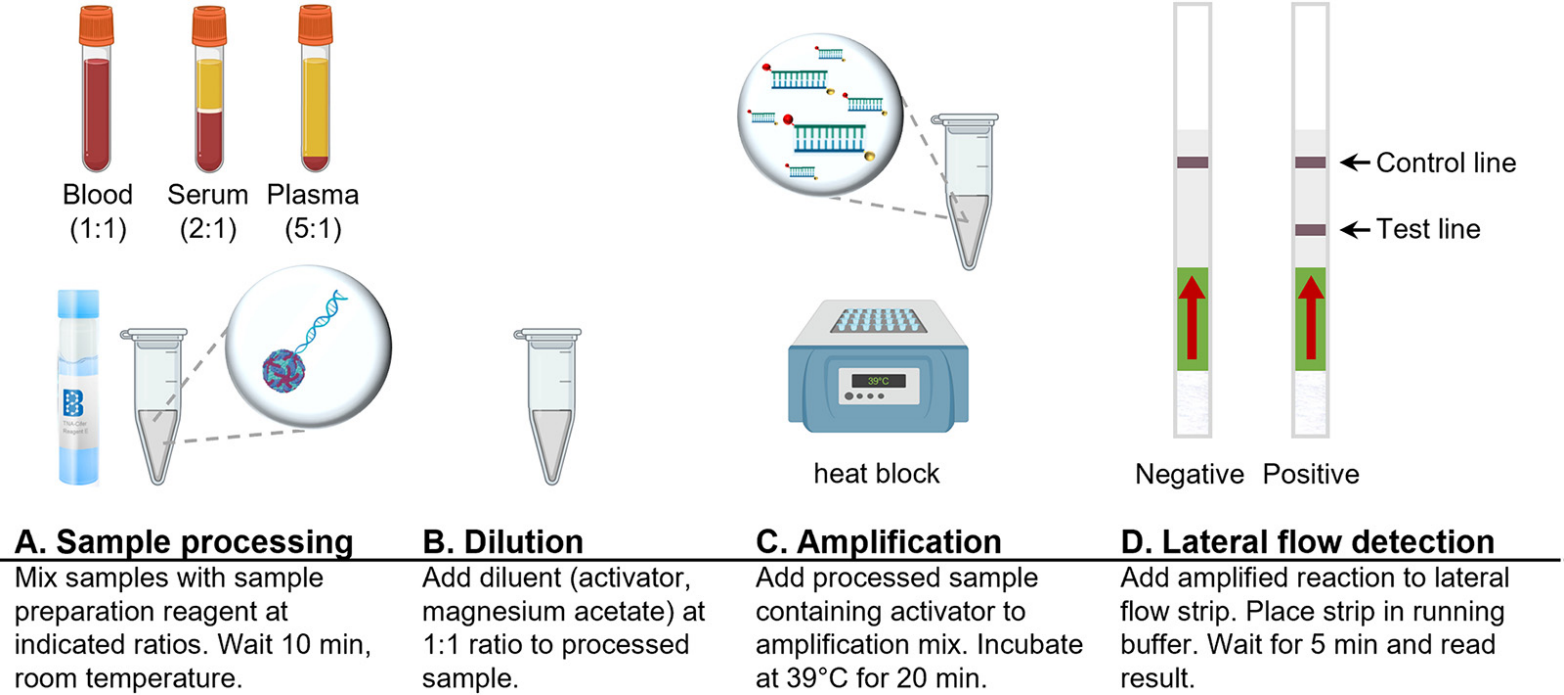
Workflow for rapid dengue serotyping tests. Shown is the basic workflow for rapid dengue serotyping tests, including sample processing, dilution, amplification, and lateral flow detection steps.

Our diagnostic test format is composed of a one-step sample preparation step (Fig. 5A and B), amplification of the NS5 target using reverse transcription-recombinase polymerase amplification (RT-RPA) (Fig. 5C), and detection using lateral flow dipsticks (Fig. 5D). In addition to specific assay components, our diagnostic tests require only a 39°C heating block and pipettes for liquid handling for completion. This study focused on evaluating the sensitivities of the four dengue diagnostic tests when used in conjunction with clinically relevant matrices by optimizing the simple sample preparation workflow for each of the clinical matrices.

### Plasmids, RNA template preparation, and oligonucleotides.

Plasmids containing the DENV-1, DENV-2, DENV-3, and DENV-4 nonstructural NS5 genes were previously described (24). For comparative RT-qPCR, a pBIC-A plasmid containing DNA encoding the anchored capsid peptide coding region (DENV-1 [GenBank accession number KF973466.1], nucleotides [nt] 95 to 436) followed by a DENV-1 NS5 region fragment (DENV-1 [GenBank accession number GU131962.1], nt 900 to 1130) was designed by and obtained from Bioneer Pacific (Victoria, Australia).

To produce RNA templates, DNA templates were linearized by restriction with XhoI (New England BioLabs Australia Pty. Ltd., Victoria, Australia), electrophoresed, and purified (NucleoSpin gel and PCR cleanup; Macherey-Nagel, Düren, Germany). RNA transcripts were generated by *in vitro* transcription according to the manufacturer’s instructions (MEGAscript T7 transcription kit; Invitrogen by Thermo Fisher Scientific Australia Pty. Ltd., Victoria, Australia). The RNA concentration was determined using a Qubit 4 fluorometer (Invitrogen by Thermo Fisher Scientific Australia Pty. Ltd., Victoria, Australia).

DENV serotype-specific primers and Nfo probes were designed from the respective DENV-1, DENV-2, DENV-3, and DENV-4 NS5 consensus sequences according to guidelines described in the TwistAmp Nfo kit protocol (TwistDX, Cambridge, United Kingdom) (24). To account for intraserotype diversity, primers and probes were designed with degenerate nucleotides to ensure that multiple strains can be detected with each serotype test.

### Viruses, cell culture, virus culture, and virus titer determination.

**(i) Viruses.** DENV strains were originally derived from the following clinical isolates: DENV-1 ET00.243 (GenBank accession number AY422468.1), DENV-1 TC861HA (GenBank accession number MF576311), DENV-2 New Guinea C (GenBank accession number M29095.1), DENV-3 ET00.209 (GenBank accession number AY422470.1), and DENV-4 ET00.288 (GenBank accession number AY422471.1).

Additional arbovirus strains used in the study were DENV-2 ET00.300 (GenBank accession number EF440433), DENV-2 Puerto Rico PR159 (GenBank accession number M19197.1), DENV-2 Timor 2004 (GenBank accession number JN568256), DENV-3 Cairns 2008 (GenBank accession numbers JN575563 to JN575580), chikungunya virus (CHIKV) Mauritius 2006, Japanese encephalitis virus (JEV) Nakayama, West Nile virus subtype Kunjin NSW 2011 (WNV_KUN_), Murray Valley encephalitis virus (MVEV) 151, yellow fever virus (YFV) 17D, and Zika virus (ZIKV) MR766.

**(ii) Cell culture.** Standard protocols for culturing Aedes albopictus clone C6/36 (ATCC CRL-1660) were used (33). In short, cells were cultured in RPMI 1640 (Thermo Fisher Scientific Australia Pty. Ltd., Victoria, Australia) with 5% heat-inactivated fetal bovine serum (FBS) (Sigma-Aldrich, New South Wales, Australia), 2 mmol/L l-glutamine (Gibco by Thermo Fisher Scientific Australia Pty. Ltd., Victoria, Australia), 100 U/mL penicillin, 100 μg/mL streptomycin, and 0.25 μg/mL amphotericin B (Sigma-Aldrich, New South Wales, Australia) (referred to as “cell culture medium”) at 28°C with 5% CO_2_. Before reaching confluence, cells were trypsinized with a 0.25% trypsin solution (Gibco by Thermo Fisher Scientific Australia Pty. Ltd., Victoria, Australia) and resuspended in fresh growth medium before plating onto a new growth surface.

**(iii) Virus culture.** Viruses were propagated in C6/36 cells at approximately 80% confluence in RPMI 1640 (Thermo Fisher Scientific Australia Pty. Ltd., Victoria, Australia) with 2% heat-inactivated FBS, l-glutamine (2 mmol/L; Gibco by Thermo Fisher Scientific Australia Pty. Ltd., Victoria, Australia), and an antibiotic-antimycotic solution (Sigma-Aldrich, New South Wales, Australia). Supernatants containing live virus were collected between 5 and 9 days after infection, clarified by centrifugation (3,000 × *g* for 10 min at 4°C), aliquoted with additional FBS to a final concentration of 10%, and frozen at −80°C.

**(iv) Virus titer determination by TCID_50_ assays and fixed-cell ELISAs.** DENV isolate concentrations were determined using the median tissue culture infectious dose (TCID_50_) assay. The cell supernatants were titrated by the TCID_50_ method on C6/36 cells in 96-well plates, with 10 wells per 10-fold dilution. The titration plates were incubated for 7 days at 28°C, cells were fixed in 20% acetone–0.02% bovine serum albumin (BSA) in phosphate-buffered saline (PBS), and replication was assessed by a fixed-cell ELISA with flavivirus envelope protein-domain II fusion peptide-reactive monoclonal antibody (mAb) BJ-6E6 (Mozzy MABs Uniquest, Queensland, Australia) (34, 35). Fixed cells were blocked for 45 min at 37°C in blocking buffer (10 mM Tris, 200 mM NaCl, 1 mM EDTA, 0.2% [wt/vol] casein, 0.05% [wt/vol] Tween 20 [pH 8.0]). The primary mAb (BJ-6E6; Mozzy MABs Uniquest, Queensland, Australia) was added to each well after the removal of the blocking buffer, and the samples were incubated at 37°C for 1 h. The plates were washed four times with PBS containing 0.05% (wt/vol) Tween 20 (PBS-T). Secondary horseradish peroxidase (HRP)-conjugated polyclonal immunoglobulin (goat anti-mouse; Dako by Agilent, Santa Clara, CA, USA) was added at its optimal concentration in blocking buffer, and the mixture was incubated at 37°C for 1 h. The plates were washed six times with PBS-T. An ABTS [2,2′-azino-bis(3-ethylbenzothiazoline-6-sulfonic acid)] (1 mM)-based substrate with 3 mM hydrogen peroxide in 0.1 M citrate–0.2 M Na_2_PO_4_ buffer (pH 4.2) was added, and the reaction was developed in the dark at RT for 1 h. The absorbance was measured at 405 nm using an EnSpire multimode plate reader (PerkinElmer, MA, USA). The titer was determined using the guidelines of Reed and Muench (36).

**(v) Mock-infected human blood, plasma, and serum.** Cultured DENVs with known viral titers (TCID_50_ per milliliter) were spiked into whole human blood (collected using K_3_ EDTA collection tubes), human plasma (Sigma-Aldrich, New South Wales, Australia), and human serum (Sigma-Aldrich, New South Wales, Australia).

### RNA purification.

Liquid-phase RNA purification was performed on viral stocks using TRIzol reagent (referred to as “TRIzol-purified” RNA) (Invitrogen by Thermo Fisher Scientific Australia Pty. Ltd., Victoria, Australia) via acidic guanidinium thiocyanate-phenol-chloroform extraction according to the manufacturer’s instructions. RNA purification using a silica-based kit (referred to as “kit-purified” RNA) was performed on mock-infected clinical samples using the NucleoSpin RNA virus minikit (Macherey-Nagel, Düren, Germany), again according to the manufacturer’s instructions (20 μL of the sample was eluted in 20 μL of nuclease-free water). All purified RNA was stored in aliquots at −80°C. Automated RNA purification (referred to as “extractor-purified” serum RNA) was performed on clinical serum samples using the IndiSpin QIAcube HT pathogen kit (Indical Bioscience, Leipzig, Germany) and the QIAcube HT automated nucleic acid extractor (Qiagen, Hilden, Germany).

### Universal dengue RT-qPCR.

RNA cycle threshold (*C_T_*) values and copy numbers were determined using universal DENV-1 to -4 (DU5 MGB2017) RT-qPCR (37, 38) for TRIzol-purified and kit-purified RNAs. RNA copy number quantification was performed by comparison to the quantified *in vitro*-transcribed RNA templates (24).

### Serotype-specific one-step dengue RT-qPCR.

*C_T_* values were determined using serotype-specific DENV RT-qPCR (39) for extractor-purified RNA.

### Rapid dengue serotyping tests.

**(i) Rapid sample processing method.** Samples were mixed with TNA-Cifer reagent E (BioCifer Pty. Ltd., Brisbane, Queensland, Australia) at different ratios depending on the sample type. Initial testing identified optimum ratios (sample to TNA-Cifer reagent E) of 5:1 for cell culture medium, 1:1 for blood, 2:1 for plasma, and 5:1 for serum, which were used for spiked samples. Rapidly processed samples were diluted in nuclease-free water at a ratio (processed sample to nuclease-free water) of 1:1 for blood, plasma, and serum and were used undiluted for cell culture medium.

**(ii) Serotype-specific DENV RT-RPA-LFD assays.** Serotype-specific DENV RT-RPA-LFD assays were performed using the TwistAmp Nfo kit (TwistDX, Cambridge, United Kingdom) and HybriDetect lateral flow strips (Milenia Biotec, Giessen, Germany). In brief, reaction mixes for the respective DENV-1 to -4 tests were prepared separately and incubated at 39°C for 20 min as previously described (24). Amplicon detection was performed using preactivated lateral flow strips (23), which were then placed into running buffer (30) for 5 min, analyzed visually, and photographed or scanned.

### Sensitivity and specificity testing.

Analytical sensitivity testing for the four serotype-specific DENV RT-RPA-LFD assays was performed using 10-fold serial dilutions of synthetic RNA transcripts containing the respective DENV-1 to -4 NS5 gene portions and 10-fold serial dilutions of kit-purified DENV isolate RNA. Analytical sensitivity testing of the four rapid dengue serotyping tests was performed using titrated virus diluted 10-fold in the clinical sample matrix. The analytical specificities of each serotype-specific DENV RT-RPA-LFD assay were determined using high concentrations of synthetic RNA transcripts (10^5^ copies/μL) from all dengue virus serotypes and kit-purified RNA of DENV-1 (ET00.243) (1.35 × 10^6^ TCID_50_/mL), DENV-2 (New Guinea C) (1.81 × 10^5^ TCID_50_/mL), DENV-3 (ET00.209) (2.89 × 10^5^ TCID_50_/mL), and DENV-4 (ET00.288) (3.31 × 10^6^ TCID_50_/mL) isolates. Specificity testing of the serotype-specific DENV RT-RPA-LFD assays was performed using TRIzol-purified RNA of the cell culture supernatants from closely related flaviviruses at high RNA concentrations (undiluted).

### Serum sample testing.

In total, 80 patient-consented serum samples collected between 2016 and 2022 were selected from the National Environment Agency (NEA) serum sample bank. The sera were archived residual samples collected from febrile patients and sent for dengue testing via a rapid dengue serology kit (SD Bioline Dengue Duo); positive samples were further serotyped using DENV RT-qPCR (39). The samples belonged to 5 groups, with each group containing 16 samples: (i) DENV-1-positive sera, (ii) DENV-2-positive sera, (iii) DENV-3-positive sera, (iv) DENV-4-positive sera, and (v) DENV-negative sera (NS1, IgM, and IgG negative by SD Bioline Dengue Duo and PCR negative by DENV RT-qPCR). All sera were retested by DENV RT-qPCR (39) using freshly extractor-purified RNA concurrently with DENV RT-RPA-LFD testing. In brief, samples were mixed with TNA-Cifer reagent E (BioCifer Pty. Ltd., Brisbane, Queensland, Australia) at a 5:1 ratio (5 μL of the sample to 1 μL of TNA-Cifer reagent E) in serum and incubated for 10 min at room temperature. Processed serum samples were mixed with an activator (140 mM magnesium acetate) at a 1:1 ratio (6 μL of the activator added to 6 μL of the processed sample). Subsequent amplification with DENV RT-RPA-LFD assays and detection using lateral flow strips were performed as described above.

### Imaging and statistical data analyses.

Lateral flow strips were imaged using the MultiDoc-It digital imaging system (UVP, Upland, CA, USA) or the Epson (New South Wales, Australia) Perfection V39 flatbed scanner, followed by analysis using ImageJ software (National Institutes of Health, Bethesda, MD, USA), as previously described (20, 30). Gray scale-converted images were used to determine the band intensity by measuring the mean gray value (limit to threshold), using a fixed-area measurement, and subtracting this value from the maximum mean gray value (255). For each test band, the average for two neighboring relative white spaces was subtracted from the band intensity to normalize the results. A sample was defined as positive if the normalized band intensity was 3 times higher than the standard deviation for the two neighboring white space values. Virus titers obtained by TCID_50_ assays were calculated using the Reed-Muench method, taking all dilution factors into account (36). Cartoons in figures were created with BioRender.

### Ethics.

All procedures involving the use of deidentified human whole blood from healthy volunteers for the purposes of developing or optimizing assays were approved by the QIMR Berghofer Human Research Ethics Committee (HREC) (reference number P2273). This HREC is constituted and operates in accordance with the National Health and Medical Research Council (NHMRC) *National Statement on Ethical Conduct in Human Research* (40). The processes used by this HREC to review multicenter research proposals have been certified by the National Health and Medical Research Council. Human serum (from male AB clotted whole blood) and human plasma (citrated) were obtained commercially from Sigma-Aldrich (New South Wales, Australia), aliquoted, and stored at −80°C. All deidentified human serum samples from the NEA serum sample bank were collected and tested with patient consent under Institutional Review Board-approved protocol number IRB003.1.
