# Supplementary material for: Rapid Diagnostic Tests for the Detection of the Four Dengue Virus Serotypes in Clinically Relevant Matrices
Source: Microbiol Spectr. 2023 Jan 23;11(1):e02796-22. doi: 10.1128/spectrum.02796-22 (PMC9927141; doi:10.1128/spectrum.02796-22)
Supplement: Supplemental file 1 — Fig. S1 to S6, Tables S1 and S2, and legend for Table S3. Download spectrum.02796-22-s0001.pdf, PDF file, 1.9 MB [file spectrum.02796-22-s0001.pdf]

Rapid diagnostic tests for the detection of the four dengue viruses in clinically-relevant matrices

***Nina M Pollak[1,2,3],** Malin Olsson[1,2,3], Madeeha Ahmed[1,3], Javier Tan[4], George Lim[4], Yin Xiang Setoh[4,5,6], Judith Chui Ching Wong[4], Yee Ling Lai[4], Jody Hobson-Peters[6], *Joanne Macdonald[2,7], *David McMillan[1,2,3]

[1]Centre for Bioinnovation, University of the Sunshine Coast, Sippy Downs, QLD, Australia
[2]DMTC Limited, Kew, VIC, Australia
[3]School of Science, Technology and Engineering, University of the Sunshine Coast, Sippy Downs, QLD, Australia
[4]Environmental Health Institute, National Environment Agency, Singapore
[5]Yong Loo Lin School of Medicine, National University of Singapore, Singapore
[6]School of Chemistry and Molecular Biosciences, The University of Queensland, St. Lucia, QLD, Australia
[7]BioCifer Pty Ltd, Brisbane, QLD, Australia

Content:

Supplementary Figure 1:

Analytical sensitivity and serotype-specificity of the DENV-2, -3 and -4 RT-RPA-LFD assays using kit-purified DENV isolate RNA. 1

Supplementary Figure 2:

Analytical sensitivity of serotype-specific DENV RT-RPA-LFD assays testing TRIzol-purified RNA of other DENV serotypes. 2

Supplementary Figure 3:

Analytical specificity of DENV RT-RPA-LFD tests testing TRIzol-purified RNA of closely related flaviviruses. 3

Supplementary Figure 4:

Sensitivity of rapid dengue serotyping tests using rapidly processed DENV isolate RNA. 4

Supplementary Table 1:

TNA-Cifer Reagent E inactivates DENV-1 (ET00.243) after a 10 min incubation at room temperature at 1:1, 2:1 and 5:1 ratio (sample to TNA-Cifer Reagent E or cell culture media). 5

Supplementary Table 2:

Optimization of rapid sample preparation with TNA-Cifer Reagent E followed by serotype-specific DENV RT-RPA-LFD assays. 6

Supplementary Figure 5:

Rapid dengue serotyping tests using rapidly processed mock human blood samples. 7

Supplementary Figure 6:

Mock samples testing using human whole blood, plasma and serum spiked with DENV isolates. 8

Supplementary Table 3:

DENV detection in patient sera (n = 80) with RT-PCR and rapid dengue serotyping tests. 9

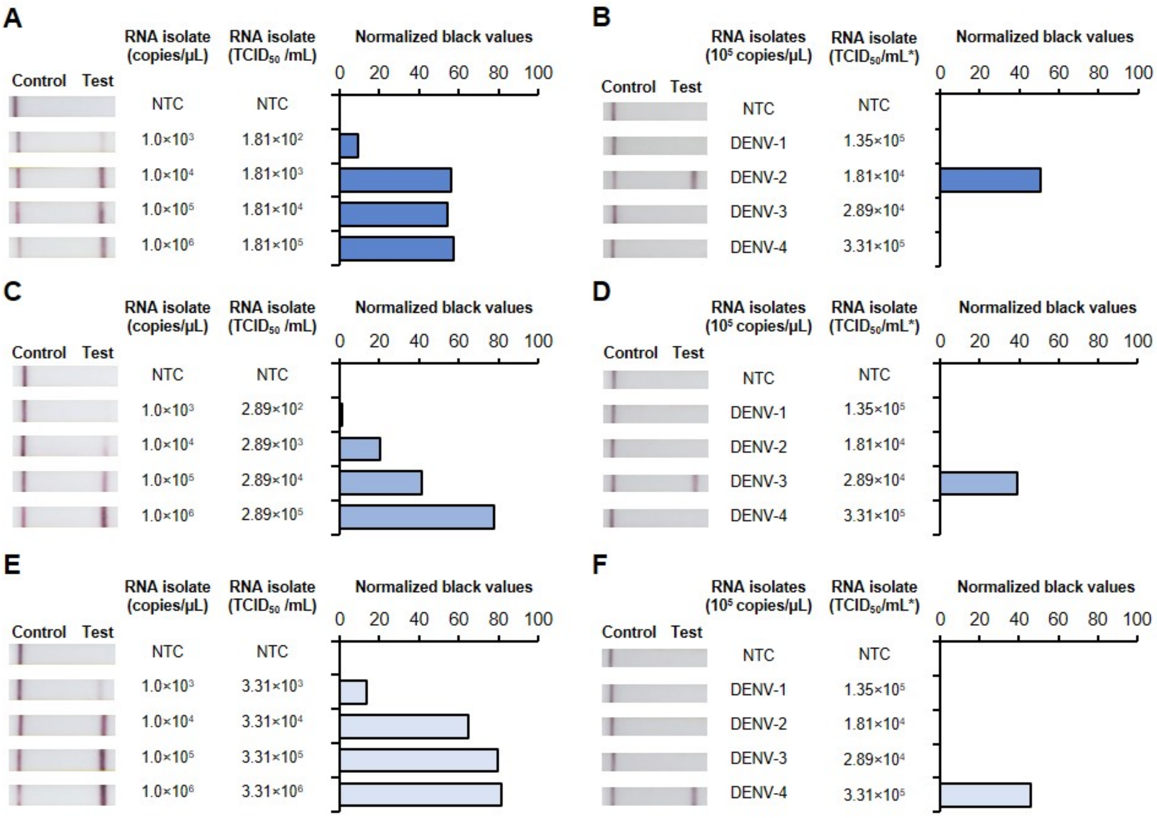

Supplementary Figure 1. Analytical sensitivity and serotype-specificity of the DENV-2, -3 and -4 RT-RPA-LFD assays using kit-purified DENV isolate RNA. Sensitivity testing used kit-purified RNA of (**A**) DENV-2 (New Guinea C), (**C**) DENV-3 (ET00.209) and (**E**) DENV-4 (ET00.288) isolates propagated with the *Aedes albopictus* mosquito cell line C6/36. Copy number (copies/µL) of kit-purified 10-fold serially diluted viral RNA was determined by universal dengue RT-qPCR using transcribed RNA as standard, which originated from a plasmid containing the Capsid peptide and NS5 region of DENV-1. Serotype-specificity was tested with kit-purified RNA of DENV-1 (ET00.243), DENV-2 (New Guinea C), DENV-3 (ET00.209) and DENV-4 (ET00.288) isolates at 10^5 copies/µL determined by universal dengue RT-qPCR with (**B**) DENV-2, (**D**) DENV-3 and (**F**) DENV-4 assays. (**A-F**) Photograph of lateral flow strips with control bands (all samples) and test bands (positive samples), nuclease-free water was used as no template control (NTC) (left). Normalised pixel density (black values) from the test displayed (right). See Figure 2C for heatmap displaying positive RT-RPA-LFD assay results per number of assay runs.

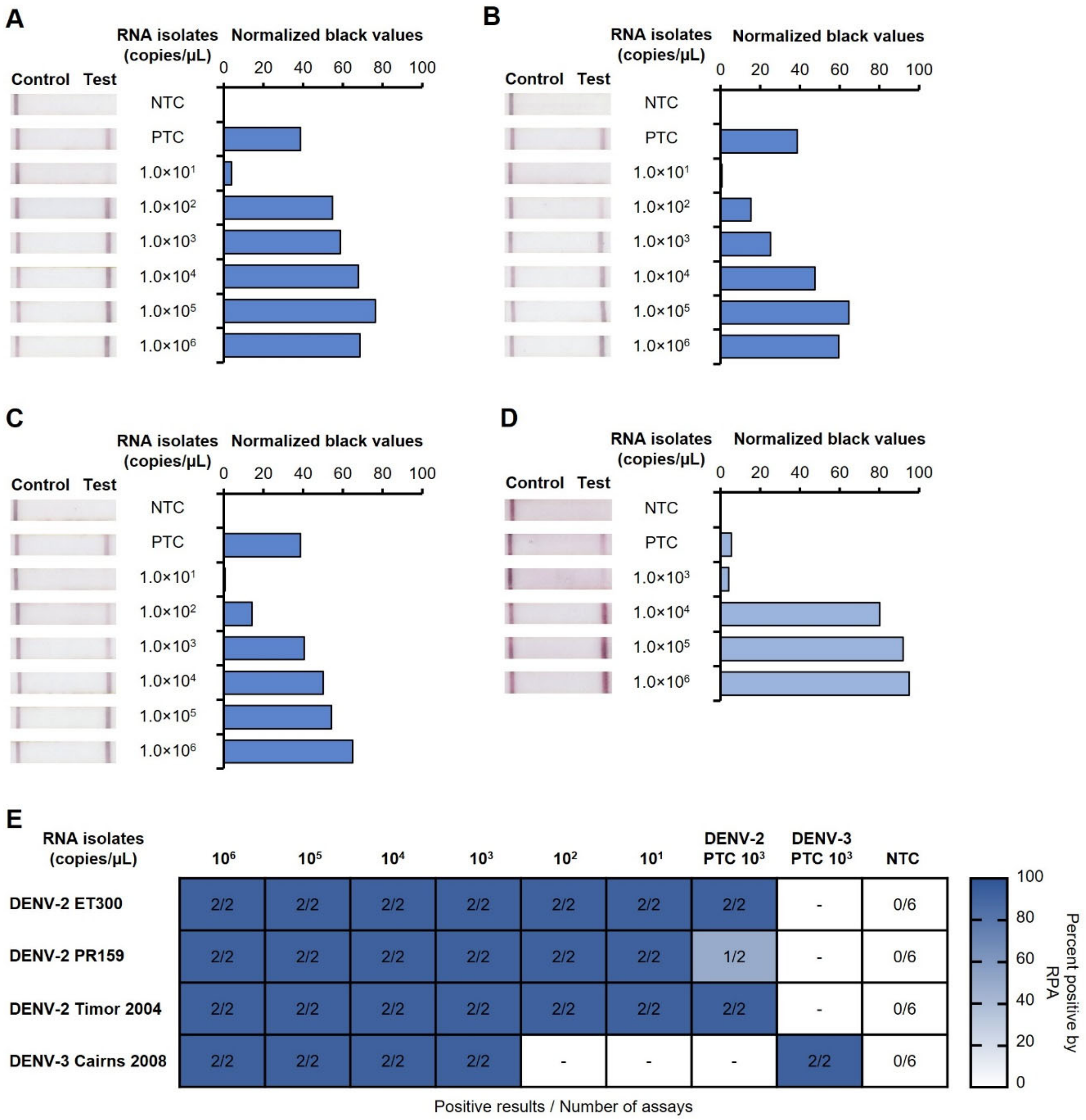

Supplementary Figure 2: Analytical sensitivity of serotype-specific DENV RT-RPA-LFD assays testing TRIzol-purified RNA of other DENV serotypes. Testing used TRIzol-purified RNA of (**A**) DENV-2 ET300, GenBank EF440433(**B**) DENV-2 Puerto Rico PR159, GenBank M19197.1, (**C**) DENV-2 Timor 2004, GenBank JN568256 and (**D**)DENV-3 Cairns 2008, GenBank JN575563-80, and transcribed RNA as positive controls at 10^3 copies/µL in the respective dengue assays. (**A-D**) Photograph of lateral flow strips with control bands (all samples) and test bands (positive samples), nuclease-free water was used as no template control (NTC) (left). Normalised pixel density (black values) from the test displayed (right). (**E**) Heatmap displaying positive RT-RPA-LFD test results for detection of specified DENV strains compared to number of assays.

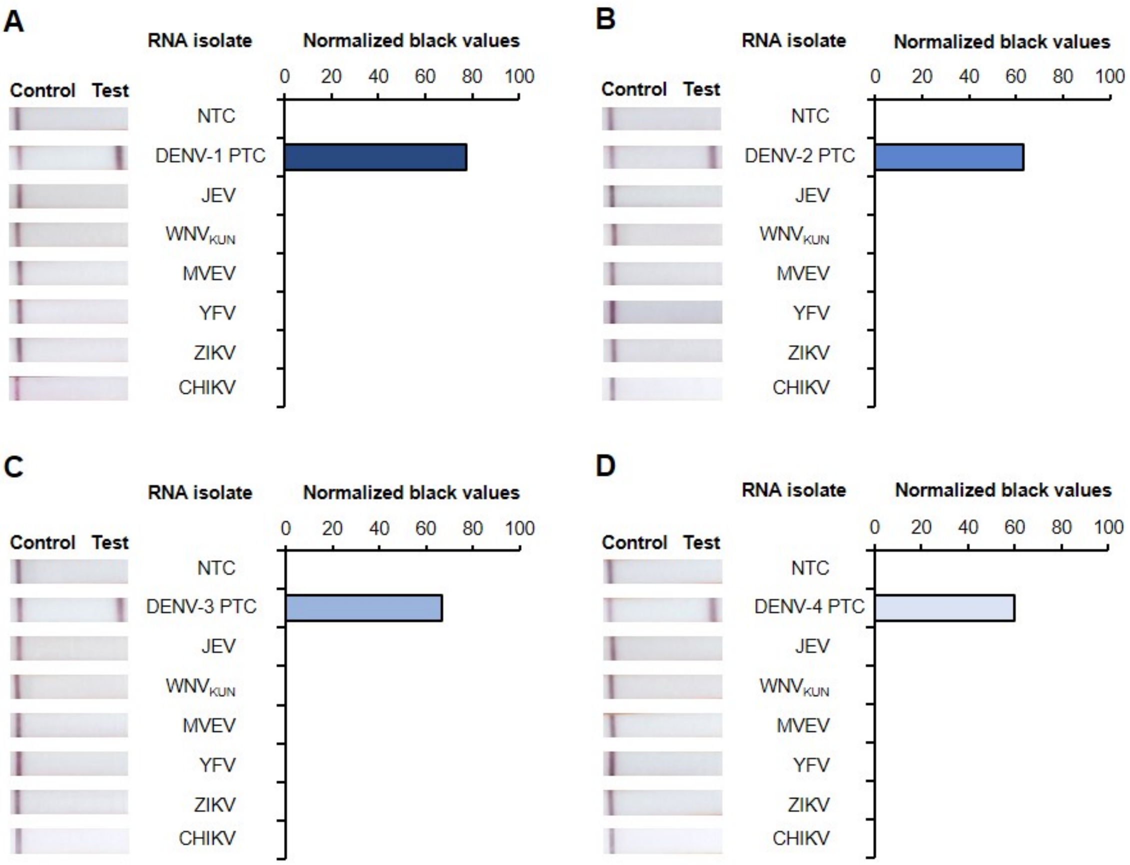

Supplementary Figure 3: Analytical specificity of serotype-specific DENV RT-RPA-LFD assays testing TRIzol-purified RNA of closely related flaviviruses. Testing used TRIzol-purified RNA of chikungunya virus (CHIKV), Japanese encephalitis virus (JEV), West Nile virus subtype Kunjin (WNV_{KUN}), Murray Valley encephalitis virus (MVEV), yellow fever virus (YFV) and Zika virus (ZIKV), and transcribed RNA of (**A**) DENV-1, (**B)** DENV-2, (**C**) DENV-3 and (**D**) DENV-4 as positive control (PTC) at 10^3 copies/µL in the respective dengue assays. Photograph of lateral flow strips with control bands (all samples) and test bands (positive samples), nuclease-free water was used as no template control (NTC) (left). Normalised pixel density (black values) from the test displayed (right). Each experiment was performed three times (see Figure 2D for heatmap displaying RT-RPA-LFD test results per number of test runs).

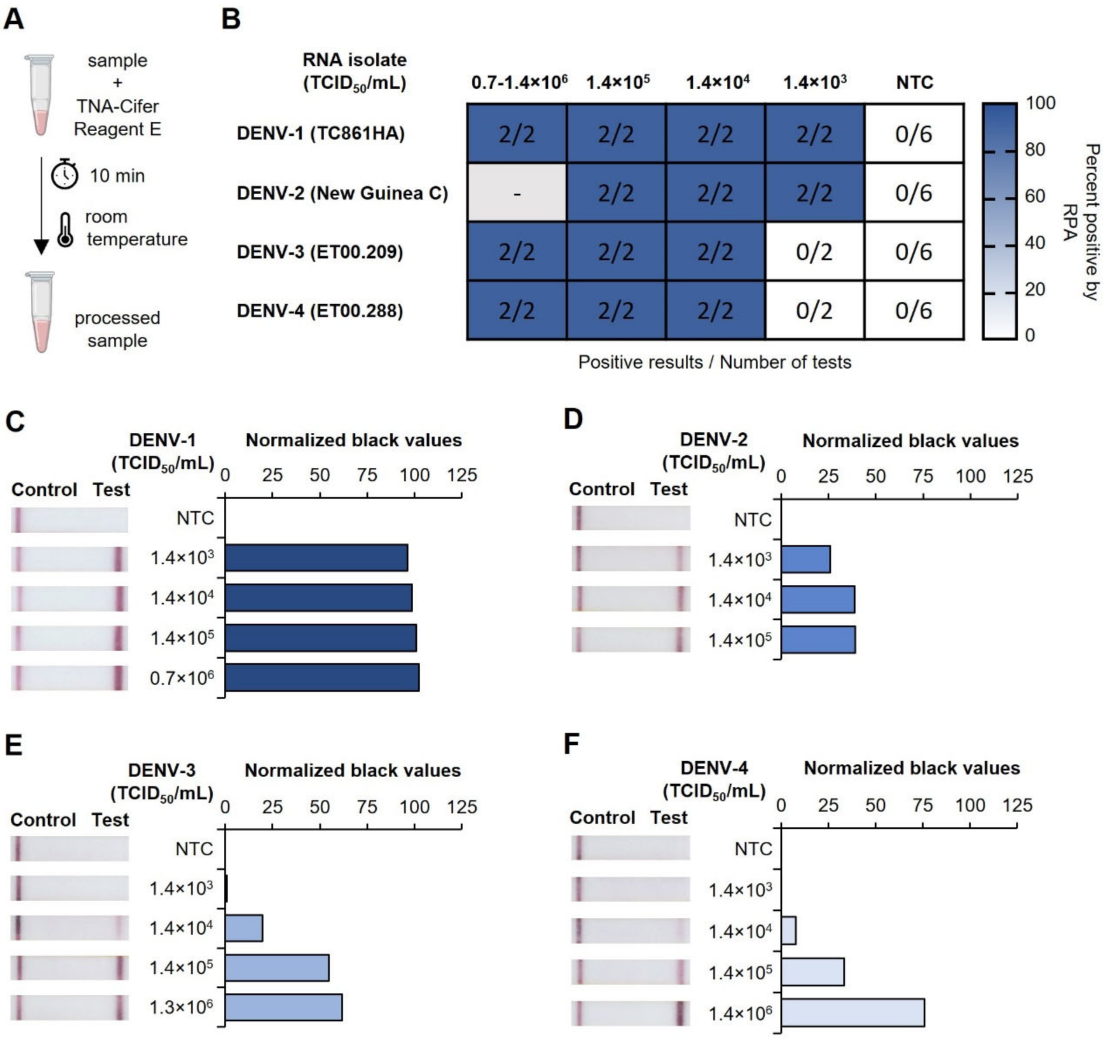

RNA isolate (TCID50/mL)	0.7-1.4×10^6	1.4×10^5	1.4×10^4	1.4×10^3	NTC
DENV-1 (TC861HA)	2/2	2/2	2/2	2/2	0/6
DENV-2 (New Guinea C)	-	2/2	2/2	2/2	0/6
DENV-3 (ET00.209)	2/2	2/2	2/2	0/2	0/6
DENV-4 (ET00.288)	2/2	2/2	2/2	0/2	0/6

Supplementary Figure 4: Sensitivity of rapid dengue serotyping tests using rapidly processed DENV isolate RNA. (**A**) Sample preparation with sample and TNA-Cifer Reagent E at a 5:1 ratio (sample to reagent). (**B**) Heatmap displaying rapid dengue serotyping test results for detection of rapidly processed DENV isolate RNA. Sensitivity testing used rapidly processed RNA (TNA-Cifer Reagent E, BioCifer) of (**C**) DENV-1 (TC861HA), (**D**) DENV-2 (New Guinea C), (**E**) DENV-3 (ET00.209) and (**F**) DENV-4 (ET00.288) isolates. Photograph of lateral flow strips with control bands (all samples) and test bands (positive samples), nuclease-free water was used as no template control (NTC) (left). Normalised pixel density (black values) from the test displayed (right).

Supplementary Table 1: TNA-Cifer Reagent E inactivates DENV-1 (ET00.243) after a 10 min incubation at room temperature at 1:1, 2:1 and 5:1 ratio (sample to TNA-Cifer Reagent E or cell culture media). Virus titre was determined by $TCID_{50}$ assays using C6/36 cells. Average ± standard deviation (n = 3).

	**DENV-1 titre (log_{10} $TCID_{50}$/mL)**		
**Ratio**	**1:1**	**2:1**	**5:1**
**No TNA-Cifer Reagent E**	6.27 ± 0.40	6.74 ± 0.38	6.18 ± 0.66
**With TNA-Cifer Regagent E**	0 ± 0	0 ± 0	0 ± 0

Supplementary Table 2: Optimization of rapid sample preparation with TNA-Cifer Reagent E followed by serotype-specific DENV RT-RPA-LFD assays. Rapid dengue serotyping tests used mock samples. Human K3 EDTA blood was spiked with DENV-4 (ET00.288) isolate resulting in 1.265 × 10^6 $TCID_{50}$/mL. Human plasma and serum were spiked with DENV-3 (ET00.209) isolate resulting in 2.25 × 10^5 $TCID_{50}$/mL. Lateral flow strip result interpretation: +++ strong visible test line; ++ visible test line; + faint visible test line; - no visible test line (confirmed by Image J analysis). All experiments included one positive control (respective transcribed DENV RNA), one negative control (no virus) and three no-template controls (nuclease-free water). Rows with bolt font and blue background highlight the preferred sample preparation and dilution ratios for the respective sample type.

**Sample type**	**Sample preparation ratio**	**Dilution ratio**	**Result interpretation**	
	Sample to TNA-Cifer Reagent E	Processed sample in nuclease-free water	Test line appearance	Positive results / Number of tests
**Blood**	1:2	1:1	+	2/2
	1:2	1:2	++	2/2
	**1:1**	**1:1**	**+++**	**3/3**
	1:1	1:2	+++	2/2
	2:1	1:1	-	0/2
	2:1	1:2	-	0/2
	5:1	1:1	++	2/2
	5:1	1:2	++	2/2
**Plasma**	**2:1**	**1:1**	**+++**	**3/3**
	2:1	1:4	+++	1/1
	2:1	1:9	+	1/1
	5:1	1:1	+	1/1
	5:1	1:4	-	0/1
	5:1	1:9	-	0/1
**Serum**	2:1	1:1	-	0/1
	2:1	1:4	++	1/1
	2:1	1:9	+	1/1
	**5:1**	**1:1**	**+++**	**3/3**
	5:1	1:4	++	1/1
	5:1	1:9	+	1/1

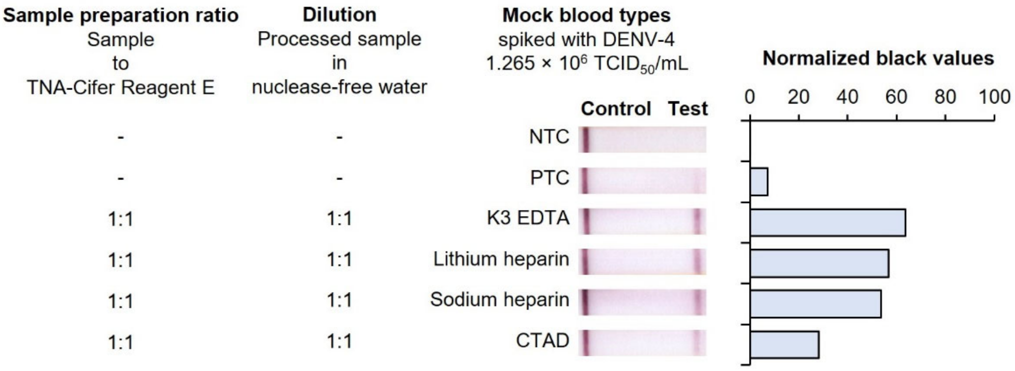

Supplementary Figure 5: Rapid dengue serotyping tests using rapidly processed mock human blood samples. Testing used rapidly processed RNA (TNA-Cifer Reagent E, BioCifer) of DENV-4 (ET288) isolate spiked into human whole blood (K3 EDTA, lithium heparin, sodium heparin and CTAD (citrate, theophylline, adenosine and dipyridamole)). Sample preparation ratio and dilution (left). Photograph of lateral flow strips with control bands (all samples) and test bands (positive samples), nuclease-free water was used as no template control (NTC) and transcribed DENV-4 RNA as positive control (PTC, at 10^3 copies/µL) (middle). Normalised pixel density (black values) from the test displayed (right).

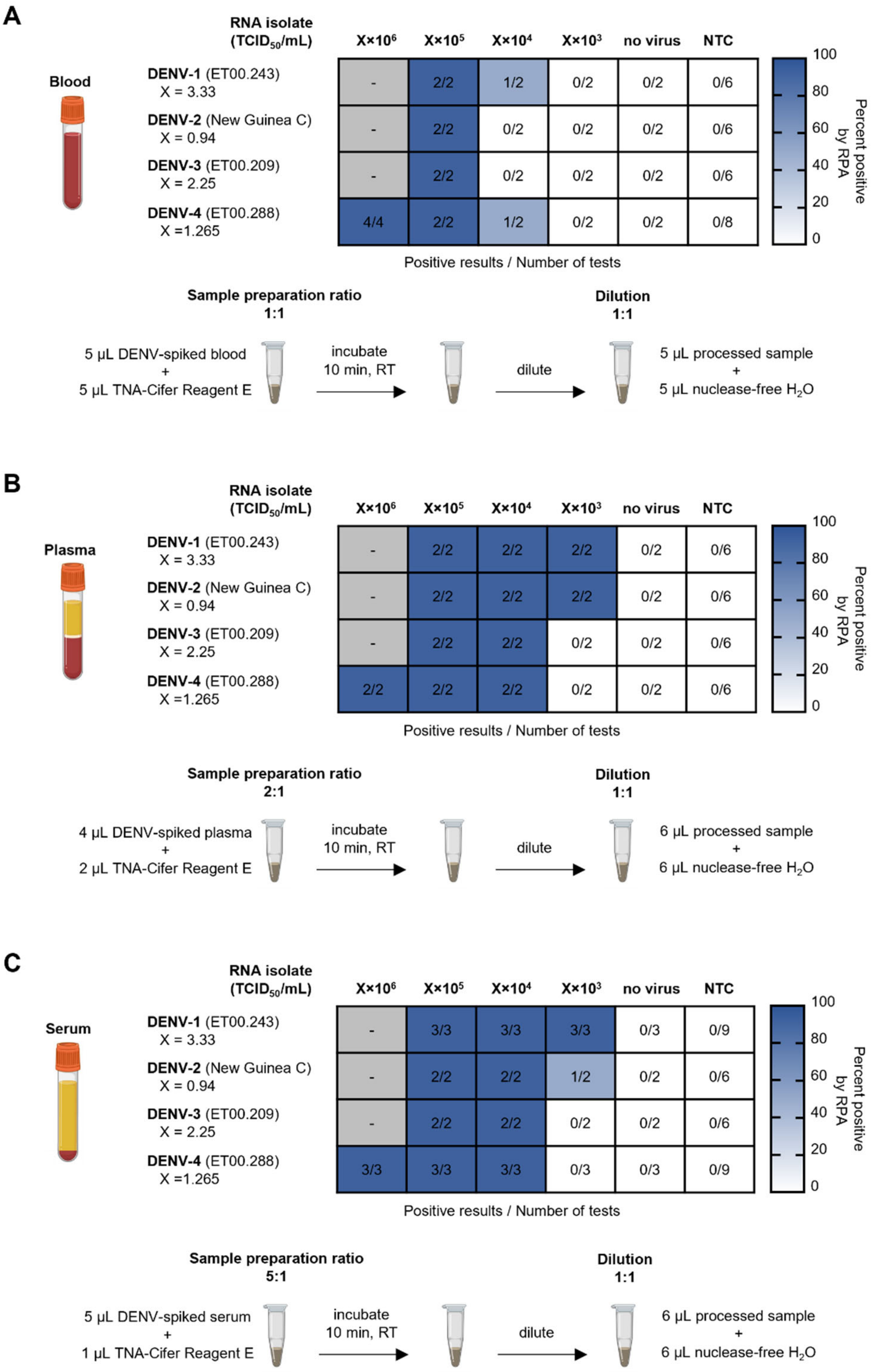

Supplementary Figure 6: Mock samples testing using human whole blood, plasma and serum spiked with DENV isolates. (**A-C**) Top: Heatmap displaying rapid dengue serotyping test calls for detection of DENV-1, -2, -3 and -4 in rapidly processed mock samples spiked with corresponding isolates. Bottom: Workflow including sample preparation and dilution of mock samples with respective ratios of reagent to sample. Sensitivity testing used rapidly processed samples (TNA-Cifer Reagent E) prepared from human (**A**) whole blood (collected with blood collection tubes containing K3 EDTA), (**B**) serum and (**C**) plasma, which were spiked with either DENV-1 (ET00.243), DENV-2 (New Guinea C), DENV-3 (ET00.209) and DENV-4 (ET00.288) isolates with respective quantified virus amounts ($TCID_{50}$/mL). Nuclease-free water was used as no template control (NTC). Blood, plasma and serum without spiked respective virus isolate were used as negative control (no virus).

Supplementary Table 3: DENV detection in patient sera (n = 80) with RT-PCR and rapid dengue serotyping tests. Displayed are Sample ID, Serotype, RT-PCR (Serotype Ct, tested concurrently with Rapid DENV serotyping tests), and Rapid DENV serotyping test results (Analyst 1, Analyst 2, Normalised black values (Image J)). Analyst 1 and 2: P+++, strong test line; P++ medium strong test line; P+, faint test line; NEG, no test line. Positive controls (DENV-1, DENV-2, DENV-3, and DENV-4 plasmid DNA at 1 × 10^6 copies/µL) showed strong test lines and no template controls (molecular-grade water) showed no test lines (data not shown).

Table provided separately for download due to dimensions.
